# Physiological and Metabolic Responses of Mongolian Horses to a 20 km Endurance Exercise and Screening for New Oxidative-Imbalance Biomarkers

**DOI:** 10.3390/ani15091350

**Published:** 2025-05-07

**Authors:** Xinzhuang Zhang, Yuanyi Liu, Lianhao Li, Wei Ma, Dongyi Bai, Manglai Dugarjaviin

**Affiliations:** 1 Key Laboratory of Equus Germplasm Innovation, Ministry of Agriculture and Rural Affairs, Hohhot 010018, China; 13470105913@163.com (Y.L.);; 2 Inner Mongolia Key Laboratory of Equine Science Research and Technology Innovation, Inner Mongolia Agricultural University, Hohhot 010018, China; 3 College of Animal Science, Inner Mongolia Agricultural University, Hohhot 010018, China

**Keywords:** Mongolian horse, exercise, oxidative imbalance, metabolome

## Abstract

The traditional horse industry has undergone a remarkable evolution, with horse racing emerging as a prominent and pivotal economic driver within the sector. Among the various breeds, Mongolian horses, renowned for their exceptional endurance and speed, occupy a significant position in the horse industry. To investigate their homeostasis mechanisms during and after a 20 km endurance exercise and identify novel oxidative-imbalance markers, we selected 12 two-year-old horses and collected blood samples at various time points before, during (at 5, 10, 15, and 20 km), and after the exercise (at 1, 2, 4, and 6 h post-exercise). These samples were analyzed for haematology, blood biochemistry, antioxidant enzyme activities, and liquid chromatography–mass spectrometry (LC-MS) metabolomics. Our results revealed significant changes in heart rate, speed, blood cells, and biochemical markers throughout the exercise. Antioxidant indicators decreased, while malondialdehyde increased, indicating oxidative imbalance post-exercise. Metabolomics analysis identified 122 differential metabolites, including uric acid and L-tyrosine, which were enriched in pathways related to energy metabolism. Uric acid and tyrosine correlated positively with serum creatine kinase, suggesting their potential as markers of oxidative-imbalance injury. These findings elucidate the mechanisms of endurance adaptability in Mongolian horses and provide a theoretical basis for mitigating oxidative imbalance, enhancing horse performance, and promoting the sustainable development of the equine industry.

## Simple Summary

This study investigated the adaptation of Mongolian horses to a 20 km endurance exercise and explored novel indicators of oxidative imbalance. Twelve young horses were selected, and their blood samples were collected before, during, and after the exercise. The results revealed changes in the horses’ heart rates, speeds, blood cells, and blood chemistry. Furthermore, post-exercise evidence of oxidative imbalance was observed, manifested by elevated creatine kinase (CK) levels and reduced catalase (CAT) activity. By analyzing the horses’ metabolites, uric acid and tyrosine were identified as potential markers for oxidative-imbalance injury. These findings enhance our understanding of Mongolian horses’ adaptation to endurance exercise and may pave the way for developing strategies to reduce stress and enhance horse performance, ultimately benefiting the equine industry.

## 1. Introduction

The traditional horse industry played a key role in transportation, mail delivery, agriculture, logging, and mining [1]. With the development of the times, the function and use of the horse have undergone a great transformation, and the horse industry has gradually evolved into a modern industry that integrates culture, products, competition, and entertainment, etc. Horse racing is the most influential industry among them, and in developed countries such as the U.S.A. and Japan, it has already become an important point of economic growth to promote the development of society [2,3]. In order to promote the transformation of China from a large country of germplasm resources to a strong country of germplasm resources, the Strategy and Action Plan for Biodiversity Conservation in China (2023–2030) lists the sustainable utilization of germplasm resources as one of the priority actions. The Mongolian horse, with its outstanding endurance and unique charm, has become one of the horse breeds that have attracted much attention in China and even around the world [4]. In 2006, the Mongolian horse was included in the List of National Livestock and Bird Genetic Resources Protection, becoming one of the first 138 national livestock and bird genetic resources protection breeds. In recent years, the Inner Mongolia Autonomous Region has vigorously developed the horse industry, created horse culture, and promoted the “Mongolian horse spirit”, making the Mongolian horse an important part of the tertiary industry in Inner Mongolia [5]. Therefore, how to protect Mongolian horse germplasm resources and promote the sustainable development of the horse industry in Inner Mongolia has become an important topic of equine scientific research.

The Mongolian horse has exhibited remarkable capabilities in long-distance races. In long-distance events, the Mongolian horse has truly shone. For example, in a grueling 15.5 km race, these horses completed the course in 24 min and 12 s [6]. This outstanding performance underscores the horse’s exceptional endurance and ability to maintain a steady pace over extended distances. Unlike some breeds that may excel in short bursts of speed, the Mongolian horse is built for the long haul. Its strong cardiovascular system and efficient energy metabolism allow it to sustain high-level performance over long periods. The economic value of the racehorse is mainly reflected in its athletic ability, and a large number of studies have shown that oxidative imbalance occurs during short-term high-intensity [7] or long-term endurance exercise [8], resulting in muscle damage and reduced athletic performance. Therefore, elucidating the homeostasis mechanism of the horse during exercise is particularly important for the horse racing industry, but at present, the metabolism of the body and the mechanism of athletic response of the Mongolian horse during exercise have not been reported.

After a horse undergoes strenuous exercise, the metabolic level of the body increases significantly, which leads to corresponding changes in blood physiological and biochemical indicators. Prolonged, high-intensity exercise may impair immune function, prompting the body to undergo an inflammatory response [9], leading to changes in routine blood indicators such as red blood cells, white blood cell counts, lymphocytes, platelet counts, and so on. A prior study performed a metabolomic analysis on horses following endurance exercise training. The findings revealed that, when subjected to a 30 km endurance load, Mongolian horses predominantly utilized aerobic fat metabolism as their energy source, accompanied by an increase in gluconeogenesis [10]. The consumption of metabolites such as carnitine and pantothenic acid increased significantly during this process [10]. Currently, most of the reports on the effects of exercise on horses focus on speed horses, and Mongolian horses, as a breed with excellent endurance, have not been reported regarding the changes in their blood metabolic mechanisms during exercise.

In this study, we analyzed the changes in blood indexes pre- and post-exercise in Mongolian horses by discussing them jointly with blood metabolomics with the aim of exploring the effects of exercise on the homeostasis mechanisms of endurance in Mongolian horses, and evaluating the level of oxidative imbalance during exercise to identify the effects of the potential biomarkers of oxidative imbalance in Mongolian horses due to exercise. To gain further insights into the metabolic pathways affected by endurance exercise in Mongolian horses, we employed KEGG (Kyoto Encyclopedia of Genes and Genomes) analysis. KEGG is a comprehensive database that integrates genomic, chemical, and systemic functional information, enabling the identification of pathways and potential biomarkers related to oxidative imbalance and endurance adaptation in horses. This analysis complements our understanding of the metabolic mechanisms underlying the adaptability of Mongolian horses to endurance exercise.

## 2. Materials and Methods

### 2.1. Selection of Experimental Animals

Twelve Mongolian horses were selected as experimental animals for this study. All of these horses were stallions, and none of them were castrated. They all originated from Xilin Gol League, Inner Mongolia Autonomous Region. Prior to the study, they underwent a six-month conditioning and training program. During this period, they were managed under a free-grazing system, which allowed them to forage freely. The age of these horses was 24 months ± 1.5 months. This sample size was determined based on previous studies in the field and was deemed sufficient to provide reliable results [11]. Prior to the commencement of the trial, these horses underwent an acclimatization period to ensure their readiness for the subsequent exercises. The horses were chosen for their suitability to participate in a high-intensity exercise program designed to investigate physiological responses. The Laboratory Animal Welfare and Ethics Committee of Inner Mongolia Agricultural University (No. NND2024009) approved all sampling procedures, which also adhered to regulatory standards.

### 2.2. Blood Sample Collection Following High-Intensity Exercise

This experiment was conducted in the pasture area of Daolunhudu Gacha Village, Daolunhudu Banner, Xilingol League, Inner Mongolia Autonomous Region. The selected horses were subjected to a 20 km high-intensity exercise program, which included a 5 km round-trip route. During the exercise, environmental conditions such as temperature, humidity, and wind speed were recorded. Body temperature was measured before and after the exercise, heart rate was monitored, and the time taken to complete the exercise was recorded to calculate the exercise speed.

To investigate the effects of exercise on the physiological functions of the horses, blood samples were collected at various time points. Specifically, samples were taken before the exercise and at 5, 10, 15, and 20 km during the exercise. Following a stationary rest period, additional blood samples were collected at 1, 2, 4, and 6 h post-exercise.

During each blood collection procedure, 10 mL of blood were carefully drawn into tubes containing the anticoagulant Ethylene Diamine Tetraacetic Acid (EDTA) and subsequently stored at a temperature of 4 °C to facilitate routine blood measurements. Simultaneously, another 10 mL of blood were collected into non-anticoagulant tubes, centrifuged to collect serum, and stored in liquid nitrogen for the determination of blood biochemical indices, antioxidant enzyme activities, and metabolomics analyses.

### 2.3. Heart Rate and Speed Monitoring

To monitor the environmental conditions of the sports grounds where the horses were situated, specific instruments were employed. The details of these instruments are provided in Supplementary Table S1. Among these, the AS-H8 Portable Digital Anemometer (Haworth, Inc., Shenzhen, China) was utilized to measure both temperature and humidity, providing crucial data on the ambient conditions during the exercise sessions. The test conditions, including wind direction, wind strength, temperature, humidity, and wind velocity, are detailed in Supplementary Table S2.

For the collection of heart rate data and measurement of the horses’ speed during exercise, the POLAR VANTAGE V wristwatch (Bonnen, Helsinki, Finland), in conjunction with the Polar H10 Heart Rate Sensor (Bonnen, Helsinki, Finland), was used. The wristwatch, equipped with advanced sensors, allowed for accurate and real-time monitoring of the horses’ physiological responses and movement parameters.

### 2.4. Hematological and Biochemical Analyses

For the hematological assessment, a blood routine examination was conducted utilizing an automatic blood routine analyzer (Shenzhen Myriad Bio-Medical Electronics Co., Shenzhen, China). This examination included the analysis of routine blood parameters such as white blood cell count (WBC), lymphocyte count and percentage, monocyte count and percentage, neutrophil count and percentage, erythrocyte count (RBC), hemoglobin (HGB), hematocrit (HCT), platelet count (PLT), mean erythrocyte volume (MCV), mean corpuscular hemoglobin (MCH), mean corpuscular hemoglobin concentration (MCHC), red cell distribution width (RDW), mean platelet volume (MPV), platelet distribution width (PDW), and plateletcrit (PCT).

Regarding the biochemical analysis of blood, an automatic biochemical analyzer (Shenzhen Myriad Biomedical Electronics Co., Shenzhen, China) was employed. The biochemical parameters analyzed included triglycerides (TG), urea (UREA), calcium (Ca), lactate dehydrogenase (LDH), aspartate aminotransferase (AST), alkaline phosphatase (ALP), total protein (TP), albumin (ALB), creatinine (CREA), glucose (GLU), total cholesterol (TC), magnesium (Mg), phosphorus (P), and glutamyl transpeptidase (GGT). Additionally, serum creatine kinase (CK) levels were measured to assess muscle damage.

### 2.5. Measurement of Blood Antioxidant Indices

The blood antioxidant indices were determined using commercially available kits supplied by the Nanjing Jianjian Institute of Biological Engineering. These indices included total antioxidant capacity (T-AOC), superoxide dismutase (SOD), catalase (CAT), glutathione peroxidase (GPx), and malondialdehyde (MDA). All measurements were conducted in strict accordance with the manufacturer’s instructions to ensure accuracy and reliability. For specific details on the indices assessed, refer to Supplementary Table S3. Each assay was performed with precision, adhering meticulously to the protocols outlined in the respective kit manuals to obtain valid and reproducible results.

### 2.6. Sample Preparation and LC-MS Analysis

For the purpose of LC-MS analysis, blood samples were collected into tubes without anticoagulant. The supernatant obtained after centrifugation of these samples was processed as follows: The supernatant was blown dry under nitrogen to remove any residual solvent. Subsequently, 100 µL of a reconstituted solution (acetonitrile:water = 1:1) was added to redissolve the dried sample. This solution was then subjected to low-temperature ultrasonic extraction for 5 min at 5 °C and 40 kHz to ensure efficient extraction of metabolites. After extraction, the sample was centrifuged at 13,000× *g* for 10 min at 4 °C to pellet any insoluble material.

The resulting supernatant was carefully pipetted into injection vials equipped with insert tubes to prevent evaporation and contamination. These prepared samples were then analyzed using liquid chromatography–mass spectrometry (LC-MS). The analysis was performed on an ultra-high-performance liquid chromatography (UPLC) tandem Fourier transform mass spectrometry (FT-MS) system, specifically the UHPLC-Q Exactive HF-X from Thermo Fisher Scientific (Waltham, MA, USA).

### 2.7. Quality Controls and Validation of LC-MS Analysis Results

To ensure the accuracy and reproducibility of the LC-MS analysis results, we implemented several quality control measures and validation steps. Raw LC-MS data were processed using Progenesis QI software (Version v3.0) (Waters Corporation, Milford, MA, USA), encompassing baseline filtering, peak identification, integration, retention time correction, and peak alignment, resulting in a clean and accurate data matrix containing retention time, mass-to-charge ratio (*m*/*z*), and peak intensity. Processed MS and MS/MS data were compared against public databases (e.g., HMDB and Metlin) and an in-house database to identify and acquire information on the metabolites present. Subsequently, preprocessed data underwent principal component analysis (PCA) and orthogonal projections to latent structures–discriminant analysis (OPLS-DA) using the ropls package (R, Version 1.6.2). Model stability was assessed through 7-fold cross-validation. Significant differential metabolites were determined based on variable importance in projection (VIP) values greater than 1 and *p*-values less than 0.05 from the OPLS-DA model and Student’s *t*-test. These quality control measures, data processing, and statistical analysis ensured the robustness and reliability of the LC-MS analysis results, providing a foundation for the identification of differential metabolites and their correlation with blood indicators.

### 2.8. Statistical Analysis of Data

The blood indices were analyzed using Excel (Version 2019) for data manipulation, while all experimental data underwent statistical analysis via SPSS 26.0 software. Results were presented as mean values, standard errors of the mean (SEM), and *p*-values, with statistical significance defined at *p* < 0.05. Data visualization was achieved using Excel.

Following metabolomics analysis by liquid chromatography–mass spectrometry (LC-MS), raw data were imported into Progenesis QI (Waters Corporation, Milford, MA, USA) for processing. This involved several steps, including baseline filtering, peak identification, integration, retention time correction, and peak alignment, culminating in the generation of a data matrix encompassing retention time, mass-to-charge ratio, and peak intensity. Concurrently, the MS and MS/MS mass spectrometry data were cross-referenced with public metabolic databases, namely the Human Metabolome Database (HMDB; http://www.hmdb.ca/, accessed on 3 January 2025) and Metlin (https://metlin.scripps.edu/, accessed on 3 January 2025), as well as Meggie’s proprietary databases, to identify and retrieve pertinent metabolite information.

Subsequently, the preprocessed data matrices were subjected to principal component analysis (PCA) and orthogonal partial least squares discriminant analysis (OPLS-DA) using the ropls package in R (Version 1.6.2). The robustness of the model was evaluated through 7-fold cross-validation. The selection of significantly different metabolites was based on variable importance in projection (VIP) values derived from the OPLS-DA model and the *p*-values from *t*-tests. Metabolites with VIP > 1 and *p* < 0.05 were deemed to be significantly different.

## 3. Results

### 3.1. Alterations in Heart Rate and Velocity of Mongolian Horses Across Varying Exercise Distances

Mongolian horses’ heart rate and speed serve as primary monitoring indices in daily exercise training, offering valuable insights into the assessment of their physical activity. As evidenced by the data presented in Table 1, the average heart rate of Mongolian horses exhibited a distinct pattern of initially increasing and subsequently decreasing with the extension of exercise distance. For details on the environmental conditions during the exercise sessions, refer to Supplementary Table S2. Specifically, the average heart rate reached its peak at 127.00 beats per minute (bpm) when the exercise distance was 5 km. Conversely, the maximum heart rate decreased from 187.08 bpm to 162.83 bpm as the exercise distance increased. Regarding speed, the average speed of Mongolian horses peaked at 19.46 km/h at a distance of 15 km, while the maximum speed showed a slight increase from 37.68 km/h to 38.68 km/h with increasing exercise distance.

### 3.2. Changes in Blood Routine Indexes of Mongolian Horses at Different Exercise Distances and Post-Exercise

Table 2 presents the alterations in routine blood indexes of Mongolian horses at various exercise distances and following exercise. For information on the instruments used to collect these data, refer to Supplementary Table S1. As depicted in Table 2, during the course of exercise, the leukocyte count of Mongolian horses gradually increased with the extension of exercise distance from pre-exercise to 20 km and was significantly higher than the pre-exercise level at 20 km (*p* < 0.05). This count continued to rise post-exercise, reaching a peak of 14.33 × 10^9^/L at 2 h post-exercise, before decreasing to 12.70 × 10^9^/L at 6 h post-exercise, yet it still remained significantly higher than the pre-exercise level (*p* < 0.05).

The lymphocyte count and percentage initially increased and then decreased. Post-exercise, these values were significantly lower than during exercise (*p* < 0.05). Specifically, the lymphocyte count was significantly higher at 5 km and 10 km compared to pre-exercise (*p* < 0.05), and then gradually declined, only to gradually increase again 2 h post-exercise.

The monocyte and neutrophil counts also followed an initial increase followed by a decrease and were significantly higher than pre-exercise levels (*p* < 0.05). These counts decreased at 2 h post-exercise but remained significantly higher than pre-exercise levels (*p* < 0.05). The monocyte count decreased 4 h post-exercise and returned to the pre-exercise level 6 h post-exercise (*p* < 0.05), while the neutrophil count also decreased but still remained significantly higher than pre-exercise levels 6 h post-exercise.

The percentage of monocytes gradually decreased with the increase in exercise distance from pre-exercise to 20 km, reaching its lowest point at 10 km, and then gradually increased, continuing to rise 1 h post-exercise. The percentage of neutrophils gradually decreased from pre-exercise to 20 km with increasing exercise distance, reaching its lowest point at 5 km, then gradually increased, and continued to rise post-exercise, reaching its highest point at 2 h post-exercise and being significantly higher than at 5 km (*p* < 0.05).

The erythrocyte count, hemoglobin, and hematocrit gradually increased from pre-exercise to 20 km with increasing exercise distance, and then increased significantly during exercise (*p* < 0.05), before gradually decreasing post-exercise and then gradually increasing again 1 h post-exercise. There were no significant changes in the platelet count and mean erythrocyte volume (*p* > 0.05).

### 3.3. Changes in Blood Biochemical Indexes of Mongolian Horses at Different Exercise Distances and Post-Exercise

Table 3 presents the alterations in blood biochemical indexes of Mongolian horses at various exercise distances and following exercise. As demonstrated in the table, statistically significant variations were observed not only in creatinine (CREA) levels but also in triglycerides (TG), glucose (GLU), and creatine kinase (CK) levels. Specifically, the creatinine level gradually increased from pre-exercise to 20 km of exercise, reaching its highest point at 20 km and being significantly higher than at the 5 km exercise distance (*p* < 0.05). Post-exercise, the creatinine level continued to rise initially but then gradually decreased, returning to a non-significant difference compared to the pre-exercise level by 6 h post-exercise (*p* > 0.05). Triglycerides (TG) and glucose (GLU) levels also exhibited characteristic patterns. Similarly, creatine kinase (CK) levels significantly increased with the extension of exercise distance, indicating muscle damage and metabolic stress, and peaked at 2 h post-exercise (*p* < 0.05). Apart from the aforementioned significant changes, the differences between the blood test results for the other parameters, including urea, calcium, lactate dehydrogenase, aspartate aminotransferase, alkaline phosphatase, total protein, albumin, total cholesterol, magnesium, phosphorus, glutamyl transpeptidase, and the remaining 12 indexes, were not statistically significant compared to pre-exercise values (*p* > 0.05). Despite these non-significant changes, these parameters generally followed a pattern of initial increase, followed by a decrease, and then a subsequent increase again post-exercise, with levels gradually returning to a steady state by approximately 2 h post-exercise.

### 3.4. Changes in Blood Antioxidant Indexes of Mongolian Horses at Different Exercise Distances and Post-Exercise

Table 4 presents the variations in blood antioxidant indexes of Mongolian horses at different exercise distances as well as following exercise. For details on the antioxidant indices assessed and the assay methods used, refer to Supplementary Table S3. Among the antioxidant indices scrutinized, catalase (CAT) emerged as the sole parameter that underwent a statistically significant alteration both during and subsequent to the 20 km endurance exercise.

CAT levels exhibited a pronounced decline from pre-exercise baselines, plummeting to their nadir at the 20 km juncture of the exercise (*p* < 0.05). Following the cessation of exercise, CAT concentrations gradually rebounded, attaining a statistically elevated level at the 6-h post-exercise timepoint relative to the 20 km benchmark (*p* < 0.05). This trajectory underscores CAT’s heightened sensitivity to the oxidative stress elicited by endurance exercise in Mongolian horses, positioning it as a potential early indicator of oxidative dysregulation.

In stark contrast, no statistically significant disparities were discerned in the levels of total antioxidant capacity (T-AOC), superoxide dismutase (SOD), glutathione peroxidase (GSH-Px), or malondialdehyde (MDA) throughout the exercise and recovery periods (*p* > 0.05). This observation further underscores the specificity of CAT’s response to exercise-induced oxidative stress, as opposed to the other antioxidant indices measured.

### 3.5. Mechanistic Changes in Blood Metabolomics of Mongolian Horses Pre- and Post-Exercise

#### 3.5.1. Comparative Analysis of PCA Pre- and Post-Exercise

**Figure 1:**
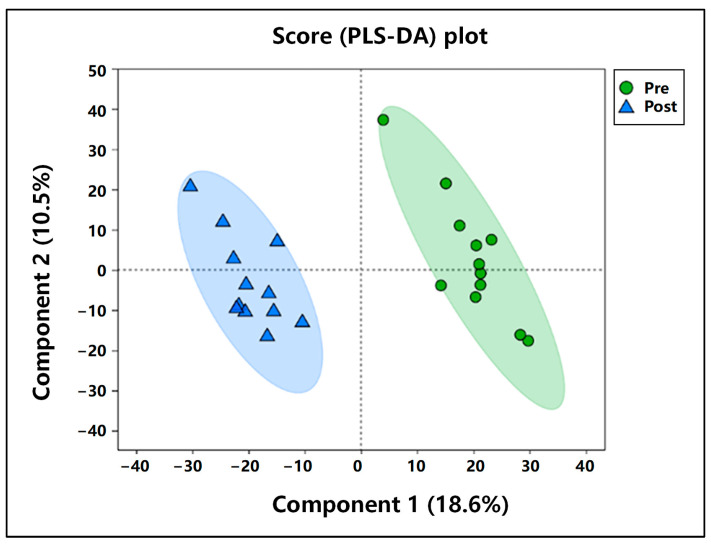
Plot of PLS-DA scores pre- and post-exercise.

As shown in Figure 1, the partial least squares–discriminant analysis (PLS-DA) model demonstrated a clear separation between the two groups of samples, suggesting that short-term high-intensity exercise exerted a significant impact on the blood metabolism of Mongolian horses. Specifically, the first principal component accounted for 18.6% of the total variance, while the second principal component contributed 10.5%.

#### 3.5.2. Differential Metabolite Identification and KEGG Pathway Enrichment Analysis in Mongolian Horses Pre- and Post-Exercise

**Figure 2:**
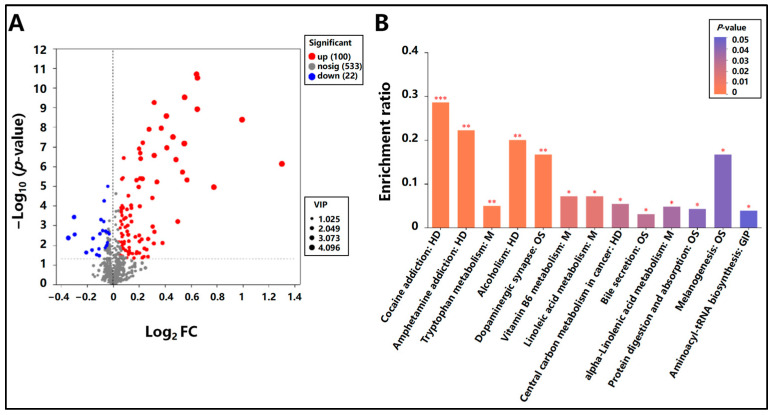
Differential metabolite identification and KEGG pathway enrichment analysis. (**A**) Volcano plot of differential metabolite statistics pre- and post-exercise in Mongolian horses. Each dot represents a metabolite, and the dot size is proportional to the VIP value; (**B**) KEGG enrichment analysis plot for differential metabolites pre- and post-exercise in Mongolian horses. The x-axis shows the pathway names, and the y-axis shows the enrichment rate. Significance levels are indicated by *, **, and *** as described above.

Figure 2 presents the identification of differential metabolites in Mongolian horses pre- and post-exercise, along with the results of KEGG pathway enrichment analysis. A comparison between post-exercise and pre-exercise samples of Mongolian horses revealed 122 differential metabolites, of which 100 were up-regulated and 22 were down-regulated (Figure 2A).

Subsequently, KEGG enrichment analysis was performed on these 122 differential metabolites. The analysis demonstrated that a total of 13 signaling pathways were significantly enriched (*p* < 0.05). Among these significantly enriched pathways were tryptophan metabolism, alcoholism, dopaminergic synapse, vitamin B6 metabolism, linoleic acid metabolism, bile secretion, alpha-linolenic acid metabolism, protein digestion and absorption, melanogenesis, and aminoacyl-tRNA biosynthesis (Figure 2B).

#### 3.5.3. Correlation Analysis of Differential Metabolites with Blood Indicators

**Figure 3:**
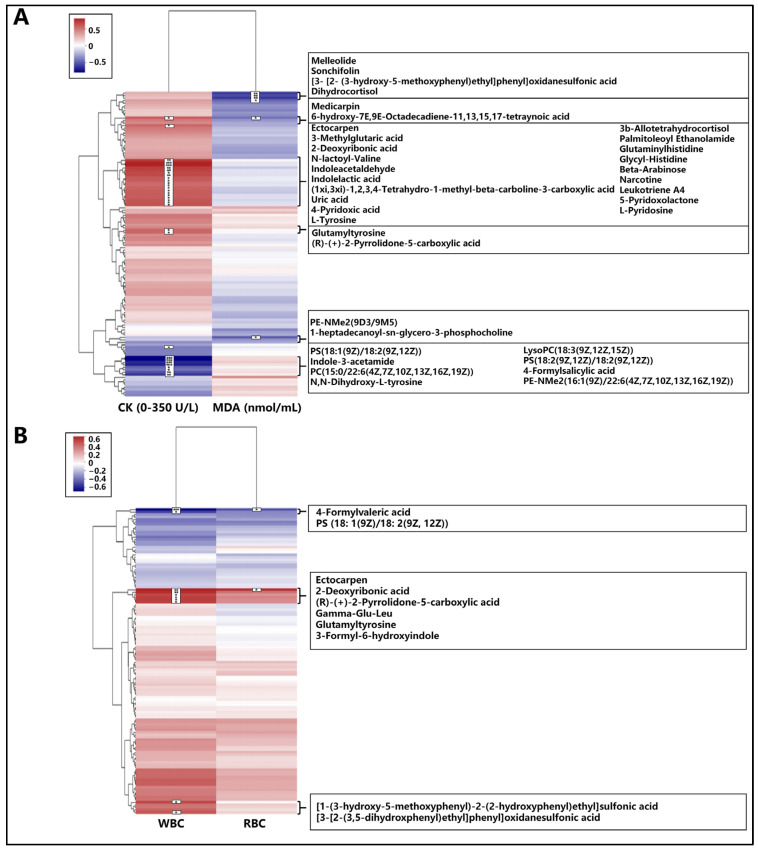
Correlation analysis of different metabolites with blood indicators in Mongolian horses. (**A**) Correlation analysis of different metabolites with serum CK and MDA. The red areas indicate a positive correlation between metabolites and CK, MDA, with deeper colors representing stronger correlations; while the blue areas indicate a negative correlation, with deeper colors representing stronger negative correlations. (**B**) Correlation analysis of different metabolites with blood WBC and RBC. Each dot corresponds to a metabolite, with significance levels denoted by *, **, and ***. The red areas indicate a positive correlation between metabolites and RBC, WBC, with deeper colors representing stronger correlations; while the blue areas indicate a negative correlation, with deeper colors representing stronger negative correlations.

To further elucidate the potential mechanisms underlying the observed metabolic changes in Mongolian horses in response to endurance exercise, we conducted a correlation analysis between the differential metabolites identified using LC-MS and various blood biochemical indices (Figure 3). This analysis aimed to pinpoint specific metabolites that might respond to alterations in locomotor adaptations, such as energy metabolism and oxidative imbalance. By correlating these metabolites with blood biochemical indices, such as serum creatine kinase (CK), malondialdehyde (MDA), white blood cell count (WBC), and red blood cell count (RBC), we aimed to identify potential biomarkers that could indicate changes in the physiological state of the horses and their adaptability to endurance exercise.

For the association analysis between differential metabolites and serum creatine kinase (CK), CK was analyzed in relation to the differential metabolites. A positive correlation with CK content was observed for the following metabolites: Medicanine, 3-Methylglutaric acid, 2-Deoxyribonic acid, Indoleacetaldehyde, Indolelactic acid, Uric acid, 4-Pyridoxic acid, L-Tyrosine, 3β-Allotetrahydrocortisol, Palmitoleoyl Ethanolamide, Glutaminylhistidine, Glycyl-Histidine, Beta-Arabinose, 5-Pyridoxolactone, L-Pyridosine, and Glutamyltyrosine. On the other hand, Indole-3-acetamide and 4-Formylsalicylic acid were negatively correlated with CK content. In the correlation analysis of malondialdehyde (MDA) with differential metabolites, Melleolide, Sonchifolin, Dihydrocortisol, and Medicanine showed a negative correlation with MDA content (Figure 3A).

Regarding the association analysis of differential metabolites with blood white blood cell count (WBC), WBC was analyzed in correlation with the differential metabolites. A positive correlation with WBC content was found for Ectocarpen, 2-Deoxyribonic acid, Gamma-GluLeu, Glutamyltyrosine, and 3-Formyl-6-hydroxyindole. In contrast, 4-Formylsalicylic acid was negatively correlated with WBC content. For the analysis of the association between differential metabolites and red blood cell counts (RBC), Ectocarpen was positively correlated with RBC content, while 4-Formylsalicylic acid was negatively correlated with RBC content (Figure 3B).

## 4. Discussion

### 4.1. Evaluation of Oxidative Imbalance

#### 4.1.1. Effects of Mongolian Horses on Heart Rate and Speed at Different Exercise Distances and Post-Exercise

Heart rate is a commonly used physiological indicator in routine sports monitoring, while speed reflects a horse’s physical state during exercise [12]. Combining these metrics allows for real-time assessment of the horse’s physiological response during acute exercise, aiding in the prevention of excessive exercise-induced decline in bodily function or physical damage. Williams [13] demonstrated that in horses, heart rate varies significantly before, during, and after exercise, with a linear relationship observed between heart rate and working speed within a certain range. Dojana’s study [14] revealed that during exercise, a horse’s heart rate ranged from 79 ± 8 to 177 ± 12 beats per minute (bpm), indicating that both exercise speed and heart rate are important indices during exercise. The results of this experiment showed a certain linear relationship between heart rate and speed during exercise, with heart rate peaking at 5 km and then gradually decreasing. Speed was highest at 15 km, and the average heart rate was elevated during this phase.

#### 4.1.2. Effects of Mongolian Horses on Blood Antioxidant Index Levels at Different Exercise Distances and Post-Exercise

Blood, as a pivotal constituent of an organism’s internal milieu, orchestrates the exchange of substances between the body and its external environment, as well as among diverse tissues. The parameters of blood serve as sentinels of an organism’s metabolic status, reflecting its physiological equilibrium or imbalance. Oxidative imbalance, a perturbation of the delicate redox equilibrium within cells, can disrupt the cellular antioxidant defense network, precipitating a decline in livestock productivity, as evidenced by prior research [15].

The pronounced decrease in catalase (CAT) levels observed during the 20 km endurance exercise in Mongolian horses underscores an augmented demand for this antioxidant enzyme to neutralize the reactive oxygen species (ROS) generated during physical exertion. CAT, a linchpin enzyme in the antioxidant defense cascade, catalyzes the dismutation of hydrogen peroxide (H_2_O_2_) into innocuous water and oxygen molecules, thereby mitigating oxidative stress and its deleterious consequences [16].

The gradual decline in CAT levels from pre-exercise baselines to their nadir at the 20 km mark, succeeded by a significant post-exercise recovery, suggests that the horses’ antioxidant defense system was taxed during the endurance trial. This reduction in CAT levels may be attributed to the enzyme’s consumption in scavenging ROS produced during muscle contraction and energy metabolism, a hallmark of strenuous physical activity. The subsequent rebound in CAT levels post-exercise heralds a recovery phase, wherein the horses’ antioxidant defenses are replenished, restoring redox homeostasis.

Despite the significant fluctuations in CAT levels, malondialdehyde (MDA) levels remained statistically invariant during and after exercise. This finding is particularly noteworthy, as MDA is widely regarded as a hallmark marker of lipid peroxidation and oxidative stress [17]. The absence of significant changes in MDA levels in this study suggests that, although oxidative stress was indeed induced by exercise, it did not surpass a critical threshold that would precipitate substantial lipid peroxidation. This resilience may be attributed to the innate endurance capabilities of Mongolian horses, which enable them to effectively buffer exercise-induced oxidative stress without succumbing to overt oxidative damage [18].

The dichotomy between the significant alteration in CAT levels and the lack of change in MDA levels further buttresses the hypothesis that CAT plays a pivotal role in the early stages of the oxidative stress response, acting as a frontline defender by scavenging ROS before they can inflict widespread damage. In this context, the decline in CAT levels may serve as an early harbinger of oxidative stress in Mongolian horses, preceding more pronounced markers like MDA, which may only show significant changes when oxidative stress has reached a more advanced stage. This insight underscores the potential utility of CAT as a sensitive biomarker for detecting oxidative stress in its nascent stages, offering valuable information for the management and welfare of performance horses.

#### 4.1.3. Effects of Blood Metabolomic Mechanisms in Mongolian Horses Pre- and Post-Exercise

In this study, metabolomics was employed to investigate the impact of acute exercise on blood metabolites in Mongolian horses. A total of 122 metabolites were identified as being differentially regulated and were significantly enriched in 13 KEGG pathways. Among these, exercise-related pathways included tryptophan metabolism, dopaminergic synapses, vitamin B6 metabolism, linoleic acid metabolism, α-linolenic acid metabolism, bile secretion, protein digestion and absorption, melanogenesis, and aminoacyl tRNA biosynthesis. Through combined analyses with creatine kinase (CK) and malondialdehyde (MDA), differential metabolites such as indoleacetaldehyde, indoleacetic acid, uric acid, L-tyrosine, and 5-pyridoxalactone were identified. Similarly, combined analyses with white blood cells (WBC) and red blood cells (RBC) revealed differential metabolites like tyrosine. Based on these findings, a comprehensive analysis was conducted to explore the potential mechanisms by which exercise regulates changes in blood metabolites.

Tryptophan Metabolism: Tryptophan, an essential amino acid for animals, interacts closely with various nutrients in its metabolic pathway, including carbohydrates, proteins, lipids, vitamins, and trace elements. Its metabolic process is influenced directly and indirectly by intestinal microorganisms, and its metabolites play crucial roles in immunity, metabolism, and neuromodulation, making them therapeutic targets for various diseases. Notably, the metabolite indole acetic acid (IAA) was significantly altered in the tryptophan metabolic pathway in a recent experiment. Exercise increased tryptophan metabolism via the tryptophan-2,3-oxidase (TDO) and indoleamine-2,3-oxidase (IDO) pathways, leading to elevated IAA production. Additionally, the study observed increased oxidative imbalance post-exercise, which may be linked to the upregulation of IAA [19]. Thus, the significant upregulation of indoleacetic acid following exercise may be associated with the activation of the tryptophan metabolic pathway and heightened oxidative-imbalance levels. After intense exercise, indole and its derivatives may also help maintain intestinal homeostatic expression by modulating pro- and anti-inflammatory cytokines [20].

Vitamin B6 Metabolism and Exercise: The metabolite 5-pyridoxalactone (PLP), an active form of vitamin B6 derived from the vitamin B6 signaling pathway enriched by the KEGG pathway, is crucial for various enzyme activities and bodily metabolism [21]. Exercise, as a physiological stimulus, positively affects body metabolism. However, muscle activity heightens the risk of vitamin B6 deficiency [22]. Insufficient vitamin B6 levels can diminish exercise capacity, reduce immunity, disrupt metabolism, decrease enzyme activity, slow redox reactions, and compromise exercise performance [23]. In the present study, Mongolian horses exhibited enrichment in the vitamin B6 metabolic signaling pathway after strenuous exercise, aligning with previous research findings [23].

Protein Digestion and Absorption: Proteins ingested into the body are continuously broken down into amino acids, peptides, and nitrogenous wastes, with nitrogenous wastes excreted in urine [24]. The most critical reaction in amino acid catabolism is deamination, which generates α-keto acids. These α-keto acids can be aminated to produce non-essential amino acids and converted into carbohydrates and lipids, a process integral to energy metabolism [25]. After strenuous exercise, the body’s energy demands surge, and energy supply may be insufficient to meet the increased need, promoting amino acid catabolism. In the protein digestion and absorption pathway, significant differences were observed in the metabolites tyrosine and proline [26].

Linoleic and α-Linolenic Acid Metabolism: This study also revealed significant enrichment in the linoleic acid metabolism and α-linolenic acid metabolism pathways. α-Linolenic acid effectively inhibits lipoprotein production in the liver and promotes their metabolism, reducing thrombosis. It possesses hypolipidemic, antithrombotic, and lipid-level-regulating abilities, as well as antiarrhythmic and antiventricular fibrillation effects, and anti-inflammatory properties [27]. Linoleic acid metabolism, a crucial metabolic pathway functioning on the basis of glycolipid metabolism, aims to degrade and consume lipids produced in the body to prevent diseases caused by excessive accumulation. It is involved in various important physiological functions, with blood glucose control being the most significant. It maintains stable blood sugar levels and supports cellular metabolic activities by activating glycolipid transporter proteins in the cell membrane to transport fatty acids into the cell [28].

Melanogenesis: Epidermal melanin, produced in the melanosomes of melanocytes, plays a vital photoprotective role [29]. Numerous factors, such as oxygen free radicals and ultraviolet light, influence melanin synthesis. In this study, the metabolite L-tyrosine was enriched in the melanogenic signaling pathway identified through the KEGG pathway. Intense exercise, such as the 20 km run performed by the Mongolian horses in this study, resulted in the production of large amounts of oxygen free radicals in the body, leading to melanin deposition [30].

Aminoacyl-tRNA Synthetases and Protein Synthesis: Aminoacyl-tRNA synthetases (aaRS) are among the earliest classes of proteins to have appeared during the evolution of life and are involved in protein biosynthesis [31]. The rates of protein synthesis and catabolism vary depending on the exercise regimen. Under standard physiological conditions, during strength training, the body adjusts the protein synthesis process, decreasing protein synthesis in non-skeletal muscle tissue and increasing it in skeletal muscle [32]. However, when exercise-induced fatigue accumulates, the rate of protein synthesis in skeletal muscle decreases, and the rate of catabolism increases, leading to a reduction in muscle protein content and thinning of muscle fibers, which significantly reduces muscle strength [33,34].

Bile Secretion and Uric Acid Metabolism: During strenuous exercise, significant differences in the metabolites cortisol and uric acid within the bile secretion metabolic pathway were identified. Research has shown that exercise intensity, rather than total workload, is the key factor contributing to the increase in blood uric acid concentrations [35]. These findings suggest that the increase in blood uric acid levels is primarily associated with enhanced purine metabolism during high-intensity exercise [36,37].

Tyrosine and Its Role in Exercise: Tyrosine is crucial for equine health and athletic performance. The effect of strenuous exercise on tyrosine levels in horses is a complex biochemical process involving multiple aspects of energy metabolism, muscle protein metabolism, and neurotransmitter synthesis. Strenuous exercise may cause damage to muscle fibers, releasing CK into the bloodstream [38]. Uric acid, tyrosine, and CK are positively correlated after exercise. There is also a positive correlation between tyrosine and WBC after exercise, as vigorous exercise may activate the horse’s immune system, leading to an increased demand for tyrosine to support the proliferation and function of immune cells [39,40].

### 4.2. Mechanisms of Endurance Adaptation Explained

#### 4.2.1. Changes in Blood Routine Indexes of Mongolian Horses at Different Exercise Distances and Post-Exercise

Prolonged and strenuous exercise can damage the local mucous membrane and impair the body’s immune capacity. Leukocytes, a crucial class of blood cells in the circulatory system, play a vital role in the body’s immune response. Monocytes, the largest type of leukocytes, and neutrophils, which account for 50–60% of the total leukocyte count in peripheral blood circulation, are highly efficient at phagocytosis and serve as the body’s first-line defense against foreign infectious agents [41]. Lymphocytes, as specific immune cells, secrete inflammatory factors, antibodies, and complement, playing a crucial role in the body’s immunity [42]. Oxidative damage to lymphocytes is a significant factor leading to the suppression of the body’s immune function [43]. The experiment revealed that the percentage of neutrophils gradually decreased as the exercise distance increased from pre-exercise to 20 km, reaching its lowest point at 5 km and then gradually increasing post-exercise, peaking 2 h post-exercise. The number and percentage of lymphocytes gradually increased with the increase in exercise distance, peaking at 10 km, and then gradually decreased post-exercise, starting to increase again 2 h post-exercise. The number of monocytes gradually increased with the increase in exercise distance and continued to rise post-exercise, reaching its highest level 1 h post-exercise.

Erythrocytes, the most abundant blood cells, are the primary carriers of oxygen transport in the body [44]. Hemoglobin, the main component of erythrocytes, binds with oxygen to transport both oxygen and carbon dioxide [45]. Erythrocyte sedimentation rate (ESR) indirectly reflects the number, size, and volume of erythrocytes [46]. The experiment showed that the number of red blood cells, hemoglobin levels, and ESR gradually increased with the increase in exercise distance, reaching their highest levels at 15 km, and then gradually decreased post-exercise, starting to increase again 1 h post-exercise. These three parameters were highly correlated, which may be related to the blood-formation mechanism. After pre-exercise training, Mongolian horses increased their total plasma protein content, leading to an increase in colloid osmotic pressure, promoting water retention in the blood circulation, and thereby increasing blood volume. Higher hemoglobin levels within the normal range are associated with better aerobic capacity. A decrease in mean corpuscular volume (MCV) reduces blood circulation resistance, positively affecting exercise. Long-term exercise causes a decrease in MCV in Mongolian horses, which is a result of exercise adaptation. Kirschvink’s study [47] proved that excessive exercise damages the body’s immune system, increasing the body’s susceptibility to infection. All these indicators suggest that the body’s immune status changes during exercise, and exercise also induces oxidative imbalance in the body.

#### 4.2.2. Changes in Blood Biochemical Indexes of Mongolian Horses at Different Exercise Distances and Post-Exercise

Under normal physiological conditions, triglycerides are broken down into glycerol and free fatty acids by lipase. However, oxidative imbalance can cause mitochondrial dysfunction within cells, leading to a decrease in β-oxidative phosphorylation in the mitochondria and causing free fatty acids to be converted back into triglycerides [48]. The experiment revealed a gradual increase in triglyceride levels as the exercise distance extended from pre-exercise to 20 km, reaching the highest level at 15 km, and then gradually decreasing post-exercise. This suggests that during exercise, the body’s triglyceride content rises, indicating the presence of oxidative imbalance. Creatinine, a by-product of muscle metabolism, is primarily excreted through glomerular filtration. Previous studies have shown that extensive exercise-induced oxidative imbalance can elevate creatinine levels [49]. In this test, creatinine levels increased from pre-exercise to 20 km as the exercise distance lengthened, reaching the highest level at 20 km, and then gradually decreased post-exercise. Creatine kinase (CK) is predominantly found in skeletal muscle and plays a direct role in cellular energy metabolism, muscle contraction, and ATP regeneration. Serum CK levels tend to rise with an increase in the intensity and frequency of physical exercise. In this experiment, CK levels gradually increased as the exercise distance extended from pre-exercise to 20 km, reaching the highest level post-exercise [50]. An elevation in CK levels indicates enhanced oxidative decomposition of amino acids for muscle energy supply, resulting in increased urea synthesis in the liver. These biochemical alterations reflect muscle tissue damage, suggesting that the training intensity was excessive and that sports-related injury has not yet fully recuperated. Notably, the severity of oxidative imbalance-induced damage demonstrates a significant correlation with alterations in relevant physiological indicators.

The inverse relationship between creatine kinase (CK) and catalase (CAT) during exercise reveals a sequential, causally linked dynamic: CAT depletion occurs first, reflecting rapid consumption to neutralize exercise-induced reactive oxygen species (ROS), while CK elevation follows later, peaking post-exercise as sarcolemmal damage from cumulative oxidative stress, mechanical strain, and ROS-triggered inflammation allows the enzyme to leak into the bloodstream [51,52]. This temporal dissociation underscores that CAT’s failure to fully counteract oxidative stress directly compromises membrane integrity, exacerbating CK release through lipid peroxidation, protease activation, and mitochondrial dysfunction, which collectively destabilize muscle structure. Consequently, CAT serves as an early sentinel of oxidative imbalance, whereas CK functions as a delayed marker of irreversible damage, forming a feedback loop where unresolved oxidative stress (evidenced by prolonged CAT suppression) amplifies mechanical injury (reflected in sustained CK elevation) [53]. This duality highlights the need for dual-marker monitoring in equine training—using CAT depletion to preempt oxidative overload and CK spikes to mitigate chronic muscle damage—while suggesting that antioxidant interventions targeting CAT preservation could attenuate downstream CK leakage and enhance post-exercise recovery.

## 5. Conclusions

Post-exercise analysis following a 20-km endurance trial in Mongolian horses revealed a significant decline in catalase (CAT) activity alongside an elevation in creatine kinase (CK) levels, indicating the onset of oxidative imbalance and the potential for sarcolemmal disruption, cellular leakage, and muscle injury. Key differential metabolites identified pre- and post-exercise included indoleacetic acid, indoleacetaldehyde, L-tyrosine, 5-pyridoxalactone, 4-pyridoxalate, 9-carbonyl, dodecanoic acid, cortisol, uric acid, and proline. KEGG pathway analysis highlighted enrichment in tryptophan metabolism, dopaminergic synapses, vitamin B6 metabolism, linoleic and α-linolenic acid metabolism, bile secretion, and energy-related pathways such as protein digestion and absorption, melanogenesis, and aminoacyl tRNA biosynthesis. Notably, uric acid and tyrosine correlated positively with serum creatine kinase (CK) pre- and post-exercise, cortisol showed a negative correlation with serum MDA, tyrosine correlated positively with white blood cells (WBC), and 4-formylsalicylic acid correlated negatively with red blood cells (RBC). These metabolites offer potential as novel biomarkers for oxidative-imbalance injury in Mongolian horses, laying a theoretical foundation for enhancing athletic performance and mitigating oxidative imbalance.

## Figures and Tables

**Table 1:** Changes in heart rate and speed of Mongolian horses at different distances and post-exercise.

Items	Travel Distance					SEM	*p*-Value
	0 km	5 km	10 km	15 km	20 km		
Average heart rate (bpm)	40.00	127.00	125.25	126.42	122.75	7.54	0.979
Maximum heart rate (bpm)	-	187.08	168.08	163.58	162.83	10.58	0.338
Average speed (km/h)	-	17.85	19.15	19.47	18.41	1.18	0.764
Maximum speed (km/h)	-	37.68	34.82	38.61	38.68	2.76	0.733

**Table 2:** Changes in blood routine indexes of Mongolian horses at different distances and post-exercise.

Items	Pre-Exercise	During Exercise				Post-Exercise				SEM	*p*-Value
		5 km	10 km	15 km	20 km	1 h	2 h	4 h	6 h		
WBC (10^9^/L)	10.13 ^B^	11.63 ^AB^	12.48 ^AB^	12.82 ^A^	13.01 ^A^	14.30 ^A^	14.33 ^A^	14.03 ^A^	12.70 ^A^	0.29	0.010
Lymph (10^9^/L)	4.59 ^BC^	5.81 ^A^	6.22 ^A^	5.55 ^AB^	5.40 ^AB^	4.12 ^CD^	3.33 ^D^	3.68 ^CD^	3.90 ^CD^	0.15	<0.001
Mon (10^9^/L)	0.54 ^CD^	0.54 ^CD^	0.53 ^D^	0.61 ^BCD^	0.65 ^ABCD^	0.81 ^A^	0.80 ^A^	0.73 ^AB^	0.71 ^ABC^	0.02	<0.001
Gran (10^9^/L)	5.00 ^D^	5.28 ^D^	5.23 ^D^	5.99 ^D^	6.96 ^CD^	9.38 ^AB^	10.21 ^A^	9.62 ^AB^	8.09 ^BC^	0.28	<0.001
Lymph (%)	46.18 ^AB^	50.20 ^A^	52.22 ^A^	46.93 ^AB^	42.47 ^B^	28.76 ^C^	23.73 ^C^	26.49 ^C^	30.84 ^C^	1.29	<0.001
Mon (%)	5.29 ^AB^	4.74 ^BC^	4.44 ^C^	4.94 ^ABC^	5.24 ^AB^	5.67 ^A^	5.44 ^AB^	5.37 ^AB^	5.66 ^A^	0.08	0.002
Gran (%)	48.53 ^BC^	45.07 ^BC^	47.58 ^C^	51.73 ^BC^	52.29 ^B^	65.58 ^A^	70.83 ^A^	68.14 ^A^	63.50 ^A^	1.26	<0.001
RBC (10^12^/L)	6.88 ^B^	7.95 ^A^	7.83 ^A^	8.10 ^A^	7.80 ^A^	6.69 ^B^	6.88 ^B^	6.93 ^B^	6.92 ^B^	0.08	<0.001
HGB (g/L)	96.92 ^B^	112.25 ^A^	112.00 ^A^	114.42 ^A^	110.25 ^A^	95.00 ^B^	95.33 ^B^	97.00 ^B^	97.55 ^B^	1.18	<0.001
HCT (%)	30.83 ^B^	35.99 ^A^	35.65 ^A^	36.93 ^A^	35.37 ^A^	29.98 ^B^	30.52 ^B^	30.65 ^B^	30.55 ^B^	0.44	<0.001
PLT (10^9^/L)	236.75	248.00	239.75	229.00	243.08	219.83	225.08	226.36	229.73	0.27	0.961
MCV (fL)	44.93	45.35	45.70	45.67	45.53	44.95	44.48	44.35	44.28	0.12	0.884
MCH (pg)	14.18	14.09	14.38	14.07	14.16	14.29	13.85	14.00	14.20	2.10	0.992
MCHC (g/L)	316.50	312.50	315.50	309.42	312.17	317.92	312.67	316.55	322.00	0.08	0.952
RDW (%)	17.75	17.98	17.83	17.85	17.96	17.71	17.88	17.79	17.69	5.47	0.994
MPV (fL)	6.65	6.73	6.67	6.92	6.98	6.86	6.77	6.77	6.65	0.05	0.798
PDW	16.05	16.05	16.40	16.28	16.08	16.18	16.20	16.05	16.20	0.05	0.816
PCT (%)	0.16	0.17	0.16	0.16	0.17	0.15	0.15	0.15	0.15	0.01	0.952

Note: WBC: White Blood Cell Count; Lymph: Lymphocyte Count; Mon: Monocyte Count; Gran: Granulocyte Count; RBC: Red Blood Cell Count; HGB: Hemoglobin; HCT: Hematocrit; PLT: Platelet Count; MCV: Mean Corpuscular Volume; MCH: Mean Corpuscular Hemoglobin; MCHC: Mean Corpuscular Hemoglobin Concentration; RDW: Red Cell Distribution Width; MPV: Mean Platelet Volume; PDW: Platelet Distribution Width; PCT: Plateletcrit. Shoulder markers with different letters in the table indicate significant differences (*p* < 0.05), while those with the same letter or no letter indicate non-significant differences.

**Table 3:** Changes in blood biochemical indexes of Mongolian horses at different distances and post-exercise.

Items	Pre-Exercise	During Exercise				Post-Exercise				SEM	*p*-Value
		5 km	10 km	15 km	20 km	1 h	2 h	4 h	6 h		
TG (mmol/L)	0.22 ^C^	0.32 ^B^	0.38 ^AB^	0.44 ^A^	0.40 ^AB^	0.20 ^C^	0.16 ^C^	0.16 ^C^	0.14 ^C^	0.01	<0.001
UREA (mmol/L)	5.64	5.59	5.69	6.23	6.28	5.96	5.87	5.83	6.01	0.07	0.218
Ca (mmol/L)	2.72	2.65	2.67	2.58	2.53	2.61	2.66	2.63	2.59	0.02	0.405
LDH (U/L)	254.57	261.10	274.62	282.25	293.71	303.14	305.38	313.64	320.20	8.73	0.635
AST (U/L)	314.36	319.19	324.96	323.18	319.43	330.91	336.25	343.02	344.15	7.86	0.991
ALP (U/L)	193.98	206.88	200.90	205.23	206.91	209.18	216.68	216.51	221.35	4.24	0.897
TP (g/L)	73.80	73.72	73.35	73.42	70.86	73.60	73.97	75.93	75.55	0.49	0.469
ALB (g/L)	29.93	30.40	30.79	30.54	30.18	31.25	31.80	30.64	31.43	0.20	0.361
CREA (μmoI/L)	57.29 ^C^	63.73 ^BC^	69.26 ^AB^	75.82 ^A^	75.94 ^A^	68.33 ^AB^	64.56 ^BC^	62.74 ^BC^	59.27 ^C^	1.04	<0.001
GLU (mmol/L)	4.22 ^B^	5.49 ^A^	5.51 ^A^	5.38 ^A^	5.23 ^A^	4.18 ^B^	4.17 ^B^	3.9 ^B^	3.99 ^B^	0.11	<0.001
TC (mmol/L)	1.58	1.54	1.56	1.60	1.53	1.57	1.62	1.62	1.63	0.02	0.970
Mg (mmol/L)	0.74	0.82	0.84	0.79	0.80	0.76	0.85	0.76	0.79	0.02	0.726
P (mmol/L)	1.50	1.66	1.70	1.75	1.76	1.51	1.60	1.79	1.75	0.03	0.352
GGT (U/L)	13.59	11.98	13.85	13.93	11.84	14.31	14.66	14.95	14.77	0.61	0.931
CK (U/L)	164.51 ^CD^	143.06 ^D^	166.17 ^CD^	199.32 ^BCD^	271.55 ^ABC^	321.43 ^A^	341.62 ^A^	297.95 ^AB^	264.41 ^ABC^	13.49	<0.001

Note: TG: Triglycerides; UREA: Urea; Ca: Calcium; LDH: Lactate Dehydrogenase; AST: Aspartate Aminotransferase; ALP: Alkaline Phosphatase; TP: Total Protein; ALB: Albumin; CREA: Creatinine; GLU: Glucose; TC: Total Cholesterol; Mg: Magnesium; P: Phosphorus; GGT: Glutamyl Transpeptidase; CK: Creatine Kinase. Shoulder markers with different letters in the table indicate significant differences (*p* < 0.05), while those with the same letter or no letter indicate non-significant differences.

**Table 4:** Changes in blood antioxidant indexes of Mongolian horses at different distances and post-exercise.

Items	Pre-Exercise	5 km	10 km	15 km	20 km	Post-Exercise 1 h	2 h	4 h	6 h	SEM	*p*-Value
T-AOC (mM)	0.52	0.50	0.48	0.46	0.44	0.45	0.48	0.51	0.52	0.08	0.975
SOD (U/mL)	18.11	18.06	17.97	17.60	17.12	17.58	17.83	18.10	18.11	0.99	0.827
CAT (U/mL)	8.84 ^A^	8.31 ^AB^	7.85 ^AB^	7.31 ^AB^	6.77 ^B^	7.24 ^AB^	7.71 ^AB^	8.48 ^AB^	8.83 ^A^	2.07	0.046
GSH-Px (U)	318.06	316.36	314.95	312.98	310.86	313.92	315.26	317.96	318.06	7.20	0.265
MDA (nmol/mL)	3.06	3.28	3.42	3.52	3.60	3.59	3.58	3.40	3.18	0.26	0.173

Note: T-AOC: Total Antioxidant Capacity; SOD: Superoxide Dismutase; CAT: Catalase; GSH-Px: Glutathione Peroxidase; MDA: Malondialdehyde. Shoulder markers with different letters in the table indicate significant differences (*p* < 0.05), while those with the same letter or no letter indicate non-significant differences.

## Data Availability

The original contributions presented in the study are included in the article/Supplementary Materials; further inquiries can be directed to the corresponding author.

## Supplementary Materials

The following supporting information can be downloaded at: https://www.mdpi.com/article/10.3390/ani15091350/s1, Table S1: Detailed Equipment Information; Table S2: Test Conditions; Table S3: Blood Antioxidant Indices and Assay Methods.
